# Different perspectives in psychiatry: how family-oriented are professionals in Germany?

**DOI:** 10.1186/s12888-024-05562-0

**Published:** 2024-02-20

**Authors:** Carolin Laser, Silke Pawils, Anne Daubmann, Antonia Zapf, Silke Wiegand-Grefe

**Affiliations:** 1 Department for Psychiatry and Psychotherapy, University Medical Center Hamburg-Eppendorf, Hamburg, Germany; 2 Department of Medical Psychology, University Medical Center Hamburg-Eppendorf, Hamburg, Germany; 3 Department of Medical Biometry and Epidemiology, University Medical Center Hamburg–Eppendorf, Hamburg, Germany

**Keywords:** Professional differences, Family-oriented mental health practice, Children of mentally ill parents, Child and adolescent mental health system, Adult mental health system

## Abstract

**Βackground:**

Children of parents with a mental illness have up to 50% chance of developing a mental illness themselves. Numerous studies have shown that preventive family-oriented interventions can decrease the risk by 40% and that professionals are a decisive factor influencing family-oriented practice. There are also substantial differences between professions in terms of their family-oriented practices. This study examines the level of family-oriented practice for different professional groups in Germany.

**Methods:**

Data were used from the baseline assessment of the two-group randomized controlled multicenter trial ci-chimps as a subproject of CHIMPS-NET, which took place from January 2020 to May 2021 in 18 clinical centers in Germany. Child and adolescent mental health systems as well as adult mental health systems took part and every professional involved in the treatment was invited to participate. Data was used from 475 mental health professionals including physicians, psychologists, psychotherapists for adults and for children and adolescents, occupational/ music/ physio/ art therapists/ (social) education workers and nursing/ education service. Family-oriented mental health practice was examined using the translated version of the Family-Focused Mental Health Practice Questionnaire (FFMHPQ) with means and standard deviations calculated for each of the 18 FFMHPQ-GV subscales. ANOVAs were computed to compare professions and significant differences were examined via post hoc analyses (Scheffé). Additionally, effect sizes were calculated (Omega squared).

**Results:**

Differences were seen between the professions in all aspects of family-oriented practice: Both regarding organizational policy and support aspects, issues concerning working with parent-clients, as well as professional skills and knowledge aspects. Psychotherapists for children and adolescents scored the highest family-oriented practices compared to all other professional groups on almost all subscales.

**Conclusion:**

This study examines the level of family-oriented practice for different professional groups in Germany. Apart from skills and knowledge about the impact of mental illness and parenting, psychotherapists for children and adolescents had the highest scores and engaged most in family-oriented practice. Psychotherapists for adults got the least workplace support for family-oriented practice but were competent providing resources and referral information to the concerned families and feel confidence working with them. Due to these results, a training need exists to improve skills and knowledge about the impact of mental illness and parenting. Additionally, there is still potential for institutional support in promoting family-oriented work.

**Trial registration:**

The CHIMPS-NET-study was registered with the German Clinical Trials Register on 2019–12-19 (DRKS00020380) and with Clinical Trials on 2020–4-30 (NCT04369625), the ci-chimps-study was registered with the German Clinical Trials Register (DRKS00026217) on 2021–08-27 and with Clinical Trials on 2021–11-04 (NCT05106673).

**Supplementary Information:**

The online version contains supplementary material available at 10.1186/s12888-024-05562-0.

## Βackground

Worldwide, 15–23% of children live with a parent with a mental illness. These children have up to 50% chance of developing a mental illness themselves [1, 2]. Numerous studies highlight that preventive family-oriented interventions can decrease the risk by 40% of developing mental disorders within the children of mentally ill parents [2–6]. Although the huge benefit of family-oriented practice is verified, there is a lack of what exactly is meant by it, why there is an immediate need for an agreed definition [7, 8]. One exemplary definition is:” Family-oriented mental health practice is defined by how professionals involve the whole family in treatment, including responding to the parenting role of the patient” [9]. Foster et al. pointed out in their definition that family-oriented practice is often conceptualized variously in child, adolescent and adult mental health services depending on how practitioner decide, who the “family” consisted of [10].

Numerous studies show that family-oriented practice is important in the child and adolescent mental health system as well as in the adult mental health system identifying and treating families with children of mentally ill parents. But it could also be shown that several preconditions are required for a successful family-oriented practice: organizational factors (policies, leadership and management of the clinical center must support family-oriented practice), routines and procedures (time and workload for family-oriented practice), professional aspects like self-reported skills and knowledge about children of mentally ill parents and family-oriented practice, beliefs about job role (e.g. is it my task working family-oriented) and perception of workplace support [11–13]. Gregg et al. summarized in their systematic review as well both aspects influencing family-oriented practice: worker and workplace factors. Worker factors included similar aspects to those found by Maybery [11, 12] and Reupert [13]: Personal attitudes like beliefs about family-oriented practice and own abilities as well as aspects regarding training and education, job role and length of service. Workplace factors included available resources, work setting, workplace support and time and workload [7].

The German mental health system bases on a complex interplay of outpatient, day-care, inpatient and complementary services, whereby the present study focuses on the inpatient setting. In an inpatient context numerous different professional groups work together and have different perspectives on the patient. There is also a subdivision in Germany for children and adolescents as well as adults. It is system inherent, that child and adolescent mental health systems work more family-oriented compared to adult mental health systems and that this influences the professionals. But both mental health systems are relevant identifying families with children of mentally ill parents, however with different focuses: While talking to parents about their children can be an important preventive factor in adult mental health systems for example [14], in child and adolescent mental health systems more attention should be paid screening the mental health of the parents upon a child’s admission to the hospital [15]. To the best of our knowledge, there are no studies so far that have examined professional differences in both child and adolescent and adult mental health systems. The review from Foster et al. (2016) found that concept and scope of family-oriented practice differs in adult and child and adolescent mental health settings [10].

Measuring all aspects of family-oriented practice Maybery et al. developed the Family-Focused Mental Health Practice Questionnaire (FFMHPQ) [16]. It has already been used in different settings and countries, and it could be shown, that there are substantial differences between professions in terms of family-oriented practice. In Australia [17] and Thailand [18] social workers scored highest overall in family-oriented practice and psychiatric nurses had lower scores on almost all aspects of family-oriented practice. Similar results could be found in Norway, where social workers scored highest and physicians scored lowest in terms of family-oriented practice [19]. Using a Chinese version of the FFMHPQ, Yao et al. stated that psychiatric nurses reported less support to families and children and psychiatric nurses were more likely to think that there was no opportunity for engagement with families [20].

Gregg et al. concluded in their systematic review that social workers may be more likely than nurses, psychologists, and psychiatrists to use a family-oriented approach in their work, particularly in terms of offering support [7]. Furthermore, they determined, that psychiatrists may incorporate more family-oriented assessment practices into their work than other professions [7].

Summarized, it can be assumed, that there are substantial differences between professions in terms of their family-oriented practices in the mental health practice. According to previous research we assume that social workers (summarized in the category “e” with occupational/ music/physio/art therapists) score the highest and nurses the lowest in family-oriented practice. Due to the fact, that there is less information about how family-oriented psychotherapists are working no clear assumption can be formulated. This study examines the level of family-oriented practice for different professional groups in Germany. As this is the first study using the FFMHPQ-GV in Germany measuring family-oriented practice no precise hypotheses can be formulated.

## Methods

### Study setting and design

Data were used from the first measurement of ci-chimps (baseline assessment), which took place from January 2020 to May 2021 in 18 clinical centers in Germany. Ci-chimps stands for “clinical implementation of CHIMPS” and is a questionnaire-based, two-group randomized controlled multicenter trial. For more information about this study see the study protocol of ci-chimps [21]. Ci-chimps is one part of a larger project, called CHIMPS-NET (children of mentally ill parents-research network), which is a stepped care model for families with a mentally ill parent. For further information about the family-oriented preventive and therapeutic interventions in CHIMPS-NET see the study protocol of the central project CHIMPS-NET [22].

### Participants

From each participating center, both the child and adolescent mental health system and adult mental health system participated. Every professional involved in the treatment of the mental health clients of the 18 clinical centers that are part of the CHIMPS-NET project were invited to participate and there were no exclusion criteria. A total response rate can’t be given because the exact number of employees for each clinical center is unknown. But we sent out 50 questionnaires per clinic, which is why we can assume at least 900 employees in total.

### Demographics and occupational information

Nine professional groups self-registered for the study including medical specialist, medical assistant, psychologist, psychotherapist, child and adolescent psychotherapist, occupational / music/physio/art therapist, (social-)education worker, nursing/education service and others. In order to take this important aspect into account and at the same time not to lose sight of the bigger picture, some professional groups were summarized. Finally, six professional groups were compared regarding their family-oriented practice: Physicians (including psychiatrists), psychologists (people, who studied psychology), psychotherapists for adults (people, who studied psychology and graduated a further education getting a psychotherapists specialized for adults (at least three years afterwards), psychotherapists for children and adolescents (people, who studied psychology or pedagogics and graduated a further education getting a psychotherapists specialized for children and adolescents (at least three years afterwards), occupational/ music/ physio/ art therapist/ (social) education worker and nursing/ education service.

Since family-oriented practice is system inherent in child and adolescent mental health systems, we decided not to compare the professions regarding their allocation to their mental health system but rather focus on the question which professions generally work in a particularly family-oriented manner.

Table 1 shows demographics and occupational information of the sample. As seen the mean age of all participants was 38.08 years, with a wide range from 19 to 68 years. 74.3% of the total 475 participants were female and 22.1% were male. Professionals working in the nursing/ education service worked the longest within their profession with 21.93 years. More than half of the participants worked in child and adolescent mental health systems.

**Table 1:** Demographics and occupational information

Characteristics	Age in years M (range)	Gender N (percentage)			Time working in… M (range) in years		Mental health system N (percentage)		
		Female	Male	Diverse / no answer	Department	Profession	Child and Adolescent	Adult	Other
All (*n* = 475)	38.08 (19–68)	353 (74.3%)	105 (22.1%)	17 (3.6%)	6.13 (0–42)	11.26 (0–42)	287 (60.42%)	168 (35.37%)	20 (4.21%)
Physicians (*n* = 120)	40.37 (25–68)	76 (63.3%)	37 (30.8%)	7 (5.8%)	5.27 (0–25)	11.71 (0–38)	64 (53.33%)	54 (45%)	2 (1.67%)
Psychologists (*n* = 99)	31.39 (24–58)	81 (81.8%)	16 (16.2%)	2 (2%)	2.52 (0–18)	4.45 (0–32)	59 (59.6%)	34 (34.34%)	6 (6.06%)
Psychotherapists for adults (*n* = 54)	39.55 (29–54)	42 (77.8%)	11 (20.4%)	1 (1.9%)	6.45 (0–28)	11.68 (0–30)	17 (31.48%)	36 (66.67%)	1 (1.85%)
Psychotherapists for children and adolescents (*n* = 56)	36.41 (25–54)	47 (83.9%)	9 (16.1%)	0	5.55 (0–20)	9.02 (0–25)	55 (98.21%)	0	1 (1.79%)
Occupational/ Music/ Physio/ Art Therapist/ (Social) education worker (*n* = 74)	42.38 (24–66)	61 (82.4%)	11 (14.9%)	2 (2.7%)	10.21 (1–39)	15.46 (1–40)	39 (52.7%)	29 (39.19%)	6 (8.11%)
Nursing/ education service (*n* = 43)	43.44 (23–60)	26 (60.5%)	15 (34.9%)	2 (4.7%)	11.03 (0–42)	21.93 (2–42)	32 (74.42%)	11 (25.58%)	0
Others (includ. Trainee) (*n* = 29)	33.50 (19–57)	20 (69%)	6 (20.7%)	3 (10.3%)	4.80 (0–30)	9.17 (0–38)	21 (72.41%)	4 (13.79%)	4 (13.79%)

### Outcome measures

**Fig. 1:**
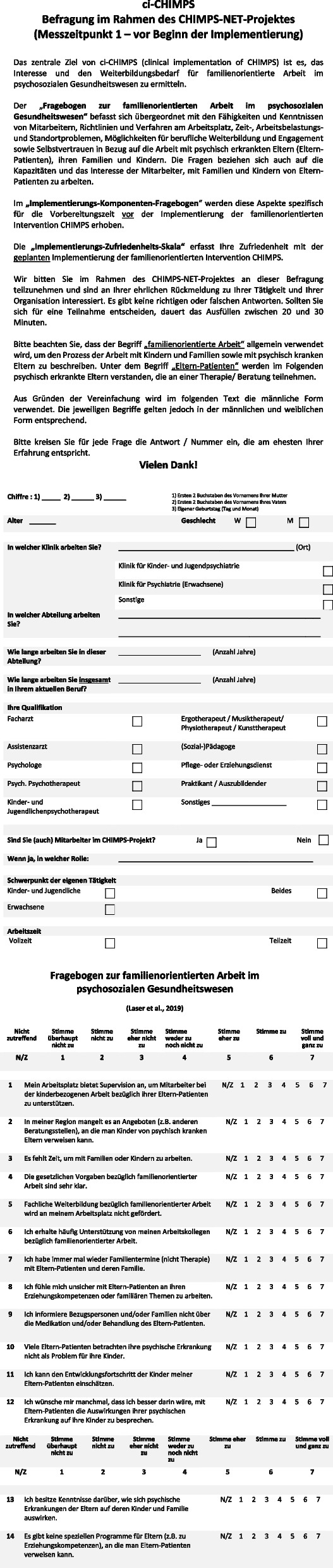

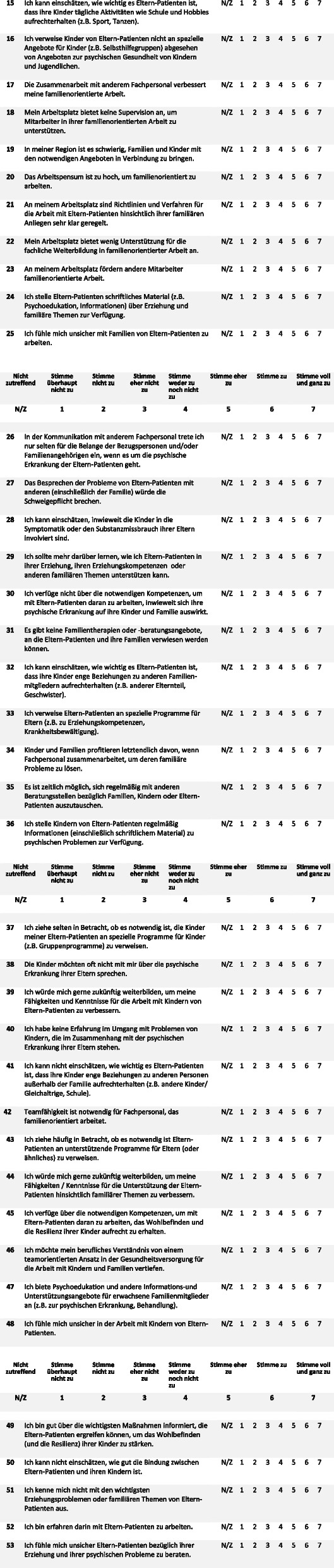
Self-constructed sociodemographic questionnaire and Family-Focused Mental Health Practice Questionnaire for Germany (FFMHPQ-GV)

Along with the FFMHPQ-GV [23], the Implementation Components Questionnaire (ICQ) [24] and Implementation Satisfaction Scale (ISS) [24] as well as a short self-constructed questionnaire was given to professionals eliciting age, gender, education, mental health service, profession type, place of service, and length of service (Fig. 1). Because of the covid-19 pandemic, an online medium was used in addition to a hard copy version. For the online assessment the platform “LimeSurvey” was used, the link was sent to all employees via the respective project employee of each center.

For the present research question, namely measuring professional differences in family-oriented mental health practice, only the FFMHPQ-GV was considered. The other two questionnaires (ICQ and ISS) focus more on the implementation process of ci-chimps and will be evaluated regarding other research questions. The FFMHPQ has 53 items with 18 subscales and measures different components of, and contributors to family-oriented practice on a 7-point Likert scale (ranging from strongly disagree to strongly agree plus not applicable). From the professional’s point of view, it records organizational and political aspects (e.g., workplace support, practice guidelines, local conditions, workload), the needs of the professionals (e.g., knowledge about families, skills about dealing with family issues, their interest in working with children, parents, and families) and what they might deliver to parents and children (e.g., psychoeducation). Additionally, it also gauges "external" factors such as health policy (see Table 2).

**Table 2:** Overview scales and items FFMHPQ-GV

Scale	Description	Included Items
Organizational Policy and Supports		
Scale 1: Workplace support	The workplace provides support (e.g. supervision) for family-focused practice	1, 18
Scale 2: Location issues	Transport and services to refer family members to are not a problem in this area	2, 19
Scale 3: Time and workload	Time or workload constraints regarding family-focused practice	3, 20, 35
Scale 4: Policy and procedures	Family-focused policy and practices are clear at the workplace	4, 21
Scale 5: Professional development	There are opportunities for professional development regarding working with families	5, 22
Scale 6: Coworker support	The support from other workers regarding family-focused work	6, 23
Working with Parent-Clients		
Scale 7: Family and parenting support	Providing resources and referral information to consumers and their families	7, 24, 36, 43, 47
Scale 8: Worker confidence	The level of confidence the worker has in working with families, parents and children	8, 25, 48
Scale 9: Support to carers and children	The level of information, advocacy and referral provided to carers and children	9, 26, 37
Scale 10: Engagement issues	The opportunity for engagement with family members	10, 27, 38
Workers Skill and Knowledge		
Scale 11: Assessing the impact on the child	How well the worker assesses the impact of the parent illness on the child/ren	11, 28
Scale 12: Training	Worker willing to undertake further training	12, 29, 39, 44
Scale 13: Skill and knowledge	Worker skill and knowledge regarding impact of parental mental illness on children	13, 30, 40, 45, 49
Scale 14: Service availability	There are programs to refer families to	14, 31
Scale 15: Connectedness	Workers assessment of parent awareness of child connectedness	15, 32, 41
Scale 16: Referrals	Referring family members to other programs	16, 33,
Scale 17: Interprofession practice	Team work and interprofessional practice	17, 34, 42, 46,
Scale 18: Parenting and mental illness	Worker skill and knowledge about the impact of mental illness and parenting	50, 51, 52, 53

The FFMHPQ-GV showed a good reliability and validity. Cronbach’s alpha coefficients were between α = 0.34 to α = 0.82, whereby 10 of the 18 subscales have a value > 0.70 and 6 subscales show at least values ranging between 0.50-0.60. Rules of thumb suggest that internal reliability is good above 0.70 [25]. In the interpretation of the results, we did not take the subscales S9 (α = 0.64), S10 (α = 0.34) and S11 (α = 0.57) into account due to the insufficient reliability with low Cronbach’s alpha values [23]. The face and content validity were estimated by a sample of clinicians and employees and was well rated [23].

## Results

All statistical analyses were performed with SPSS (version 26.0 or newer). For the participants who had less than 5% missing values, the missing values were replaced with the Expectation–Maximization-algorithm [26]. From the 594 cases, finally, data was used from 475 mental health professionals.

### Professional differences

To compare different professional groups regarding their family-oriented mental health practice in Germany, means and standard deviations (SD) were calculated for each of the 18 FFMHPQ-GV subscales (dependent variable). ANOVAs were computed for the professions (independent variable) and significant differences were examined via post hoc analyses (Scheffé).

Additionally, effect sizes were calculated (Omega squared, Ω2) to estimate, whether the effect is large, small or medium, regardless of the significance. Omega squared is the effect size of choice for ANOVAS [25]. Values with Ω2 = 0.01 represent according to Cohen a small effect size, with Ω2 = 0.06 a medium effect and Ω2 = 0.14 a big effect [25].

Table 3 shows the level of family-oriented practice for different professional groups in Germany.

**Table 3:** Professional differences regarding the FFMHPQ-GV

									Sign. Post-hoc -tests Scheffé	Effect size Omega squared (Ω^2)^
		a	b	c	d	e	f	g		
	All (*n* = 475)	Physicians (*n* = 120)	Psychologists (*n* = 99)	Psychotherapists for adults (*n* = 54)	Psychotherapists for children and adolescents (*n* = 56)	Occupational/ Music/Physio/Art Therapist/ (Social-)educ. worker (*n* = 74)	Nursing / education service (*n* = 43)	Others (includ. Trainee) (*n* = 29)		
	M(SD)	M(SD)	M(SD)	M(SD)	M(SD)	M(SD)	M(SD)	M(SD)		
Organizational policy and supports										
Workplace support (scale 1)	3.90 (2.09)	4.03 (2.08)	3.68 (2.06)	3.24 (1.88)	4.97 (1.74)	3.77 (2.15)	4.05 (2.35)	3.39 (1.95)	d > b,c	.057
Location issues (scale 2)	3.53 (1.48)	3.28 (1.29)	3.45 (1.54)	3.96 (1.30)	3.79 (1.47)	3.68 (1.61)	3.64 (1.40)	2.89 (1.83)		.036
Time and workload (scale 3)	3.22 (1.39)	3.16 (3.03)	3.03 (1.49)	3.03 (1.17)	3.79 (1.29)	3.44 (1.46)	3.21 (1.29)	2.71 (1.24)		.054
Policy and procedures (scale 4)	2.84 (1.36)	2.66 (1.26)	2.58 (1.42)	2.88 (1.12)	2.84 (1.38)	3.12 (1.47)	3.16 (1.32)	3.29 (1.47)		.027
Prof. development (scale 5)	4.28 (1.71)	4.34 (1.75)	4.21 (1.85)	4.20 (1.60)	4.84 (1.57)	4.41 (1.56)	4.00 (1.75)	3.43 (1.53)	d > g	.058
Coworker support (scale 6)	4.19 (1.60)	4.22 (1.49)	4.18 (1.65)	4.11 (1.45)	4.47 (1.51)	4.22 (1.76)	3.98 (1.68)	3.96 (1.85)		-.001
Working with Parent-Clients										
Family and parenting support (scale 7)	3.56 (1.59)	4.03 (1.43)	3.26 (1.52)	3.98 (1.19)	4.33 (1.30)	3.27 (1.67)	2.61 (1.54)	2.48 (1.89)	a > b,f,g d > b,e,f,g c > f,g	.218
Worker confidence (scale 8)	4.31 (1.78)	4.22 (1.80)	4.16 (1.85)	4.94 (1.28)	5.06 (1.72)	4.21 (1.59)	4.04 (1.73)	3.24 (2.12)	c > g d > g	.072
Support to carers & children (scale 9)	3.80 (1.81)	4.38 (1.77)	3.32 (1.79)	4.17 (1.33)	3.95 (2.13)	3.64 (1.75)	3.69 (1.42)	2.73 (2.01)	a > b,g	.087
Engagement issues (scale 10)	3.42 (1.29)	3.56 (1.22)	3.08 (1.38)	3.59 (1.26)	3.99 (1.09)	3.45 (1.16)	3.32 (1.15)	2.62 (1.64)	a > g d > b,g	.062
Worker skill and knowledge										
Assessing the impact on the child (scale 11)	3.62 (1.66)	3.87 (1.55)	3.45 (1.80)	3.15 (1.34)	4.13 (1.71)	3.63 (1.54)	3.76 (1.60)	2.74 (1.88)	d > g	.040
Training (scale 12)	4.81 (1.54)	4.93 (1.68)	4.83 (1.60)	5.08 (.93)	4.42 (1.46)	4.69 (1.48)	4.87 (1.53)	4.75 (1.91)		.065
Skill and knowledge (scale 13)	4.38 (1.32)	4.49 (1.22)	4.19 (1.39)	4.76 (1.02)	4.98 (1.07)	4.44 (1.27)	3.94 (1.15)	3.15 (1.71)	a > g b > g c > g d > b,f,g e > g	.020
Service availability (scale 14)	4.14 (1.61)	4.10 (1.58)	3.88 (1.84)	4.65 (1.24)	4.25 (1.62)	4.46 (1.42)	3.98 (1.32)	3.41 (1.90)		.049
Connectedness (scale 15)	4.49 (1.57)	4.67 (1.49)	4.11 (1.85)	4.54 (1.12)	4.67 (1.86)	4.62 (1.45)	4.71 (1.15)	3.93 (1.52)		.029
Referrrals (scale 16)	3.36 (1.94)	3.87 (1.73)	2.78 (1.93)	3.58 (1.71)	4.12 (1.87)	3.32 (2.15)	2.82 (1.62)	2.24 (2.09)	a > b,g d > b,g	.130
Interprofession practice (scale 17)	5.82 (.95)	5.96 (.72)	5.80 (1.05)	5.79 (.62)	5.97 (.70)	5.75 (1.05)	5.77 (.86)	5.27 (1.70)		.027
Parenting mental illness (scale 18)	3.04 (1.27)	3.09 (1.35)	3.24 (1.28)	2.94 (1.01)	2.49 (1.16)	2.93 (1.16)	3.57 (1.21)	2.90 (1.56)	b > d f > d	.092

Table 3 shows that in all aspects of family-oriented practice differences were seen between the professions: Both regarding organizational policy and support aspects (S1, S5), issues concerning working with parent-clients (S7, S8, S9, S10), as well as professional skills and knowledge aspects (S11, S13, S16, S18). Overall, psychotherapists for children and adolescents had almost everywhere the highest scores compared to the other professional groups.

### Organizational policy and support

Psychotherapists for children and adolescents got the most workplace support (S1) and opportunities for professional development (S5), psychotherapists for adults got the least workplace support (S1).

### Working with parent-clients

Psychotherapists for children and adolescents, physicians and psychotherapists for adults had the highest scores for the subscale “family and parenting support (S7)”. The lowest scores had nurses and other professional groups.

Regarding the subscale “worker confidence (S8)" psychotherapists for adults and children and adolescents had the highest scores, other professional groups had the lowest scores.

### Worker skill and knowledge

Psychotherapists for children and adolescents, psychotherapists for adults and physicians had the highest scores on the subscale “skill and knowledge (S13)”. Psychotherapists for children and adolescents and physicians referred family members to specific programs the best (S16). Nursing / education service and psychologists had the highest scores at the subscale “parenting mental illness (S18)”, psychotherapists for children and adolescents had the lowest score.

Considering the effect size a small effect with values with Ω^2^ ≤ 0.06 was found for the subscales “workplace support (S1)“, “location issues (S2)“, “time and workload (S3)”, “policy and procedures (S4)”, “prof. development (S5)“, “coworker support (S6)“, “assessing the impact on the child (S11)”, “skill and knowledge (S13)”, “service availability (S14)“, “connectedness (S15)“ and “interprofession practice (S17)“.

A medium effect with values with Ω^2^ ≤ 0.14 was found for the subscales “worker confidence (S8)“, “support to carers & children (S9)”, “engagement issues (S10)“, “training (S12)“, “referrrals (S16)“ and “parenting mental illness (S18)”.

A big effect with value with Ω^2^ ≥ 0.14 was found for the subscale “family and parenting support (S7)”.

## Discussion

It could be clearly shown that there are relevant differences regarding the level of family-oriented practice for different professional groups in Germany. These results are consistent with previous studies [17–20].

### Profession

Psychotherapists for children and adolescents had almost everywhere the highest scores and engaged most in family-oriented practice. They got the most workplace support (S1) and opportunities for professional development (S5), had high worker confidence (S8), skill and knowledge (S13) and referred family members to specific programs the best (S16). Remarkable is, that psychotherapists for children and adolescents had the lowest score at all at the subscale “parenting mental illness (S18)”. Nursing / education service and psychologists had the highest scores here.

Psychotherapists for adults got the least workplace support for family-oriented practice (S1). Nevertheless, regarding their work with parent-clients they had high scores for subscales”family and parenting support (S7)” and “worker confidence (S8)", which means that they provided resources and referral information to the concerned families and felt confidence working with them. Additionally, they perceived skills around being able to assess the impact of parental mental illness on children and had knowledge around the impact of parental mental illness on family members including children (S13).

Other professional groups had on many scales the lowest scores, e.g., the subscales “skill and knowledge (S13)” and “worker confidence (S8)".

Physicians had high scores for the subscales “family and parenting support (S7)” and “skill and knowledge (S13)”.

Overall, the most relevant difference was found for the subscale “family and parenting support (S7)”, which was also reflected in the effect size, which showed a large effect (Ω^2^ = 0.218): Psychotherapists for children and adolescents, physicians and psychotherapists for adults had the highest, nurses and other professional groups the lowest scores.

One possible explanation for these professional differences could be training. While training to become a child and adolescent psychotherapist focuses on learning how to work in a family-oriented way, other professional groups such as nurses or doctors have almost always focused on the individual patient and the acquisition of knowledge, up to know. In order to increase the likelihood of identifying children of mentally ill parents at an early stage, it is important that numerous professionals are sensitized for this issue.

### International comparison

International comparisons show that social workers had almost everywhere the highest scores [17–19] and psychiatric nurses the lowest scores [17, 18, 20]. Many relevant differences could be found between the professional groups for the subscale "time and workload (S3)" in Thailand [18], Norway [19] and China [20], but not in Germany. Likewise, it could not be confirmed for Germany that social workers scored the highest and nurses the lowest. One possible explanation for this could be, that we summarized social workers with other professional groups.

### Mental health system

As Gregg et al. concluded in their systematic review both worker and workplace factors influence family-oriented practice [7]. Thus, making a comparison of the professional groups regarding their family-oriented practices, you can't avoid considering the mental health systems their working in.

In our sample psychotherapists for children and adolescents worked in child and adolescent mental health systems, where family-oriented practice is system inherent. For this system working with families has traditionally been seen as a particular area of expertise, both the organization, the structures and the professionals are oriented towards a family-oriented practice. It could therefore be expected that they report more activity in this area than others.

This is in contrast with the adult mental health system, where the focus is almost more on the individual patient so far. According to Maybery [12] the most important predictor for family-oriented practice by adult mental health professionals were their perceived skill, knowledge and confidence for working with parental mental illness [12]. Tchernegovski et al. [27] found in their study, that for some mental health clinicians it is difficult to maintain a dual focus that incorporates the needs and experiences of parents and their children. Such feelings could lead professionals to believe that the needs of parents, conflict with the needs of children [27].

### Implications

Due to these results the following implications for practice can be derived: Even when psychotherapists for children and adolescents had almost everywhere the highest scores, it exists a training need, as shown by the low values for the subscale “parenting and mental illness (S18)”, to improve their skills and knowledge about the impact of mental illness and parenting. Additionally, it would be important, that organizational structures support more the early detection and treatment of families with children of mentally ill parents.

Considering the current findings, we would also recommend a specific training to psychotherapists for adults and psychologists regarding family-oriented practice to ensure an early detection and treatment of families with children of mentally ill parents.

Besides training for professionals, it seems like there is potential for institutional support regarding the adult mental health system. It needs an organizational culture which generally promotes family-oriented work and makes time and sources available.

Furthermore, these results point out that it could be helpful to sensitize other professional groups regarding family-oriented practice, who do not have specialized education, but still work with families. It’s not necessary and reasonable having a deep knowledge about parental mental illness, but a training in basic family-oriented practice would be a benefit.

### Strengths and limitations

The key strength of the current study is that it’s the first time using the FFMHPQ-GV examining professional differences in both child and adolescent and adult mental health systems in Germany. Remarkable is especially the heterogeneity and completion of the professions (all major professions are represented) as well that child and adolescent and adult mental health systems from all over Germany were included. Another force is that the FFMHPQ has been used in other countries, whereby it is possible comparing the outcomes. It would be exciting using the questionnaire in other settings and samples to evaluate, audit and improve family-oriented practice.

But there are also weaknesses with this research that limit the generalizability of the findings: Due to the insufficient reliability with low Cronbach’s alpha values, we could not take the subscales S9 (α = 0.64), S10 (α = 0.34) and S11 (α = 0.57) into account within the interpretation [23]. Another limitation is that the family-oriented practice data relied on professional’s self-assessment of practice in this study. This may not reflect what professionals actually do in practice and might be biased.

Moreover, when considering the results, it should also be taken into account, that the employees work in the 18 clinics that are part of the Chimps-net network. It can therefore be assumed, that there is a selection bias because they are already interested in this topic of children of mentally il parents. Similarly, usually only those people take part in studies who are interested in research and this topic.

## Conclusion

Differences were seen between the professions in all aspects of family-oriented practice. Psychotherapists for children and adolescents had almost everywhere the highest scores and engaged most in family-oriented practice except their skills and knowledge about the impact of mental illness and parenting. Psychotherapists for adults got the least workplace support for family-oriented practice but were competent providing resources and referral information to the concerned families and felt confidence working with them. Other professional groups had on many scales the lowest scores. Due to these results, it exists a training need to improve skills and knowledge about the impact of mental illness and parenting. Additionally, there is still potential for institutional support in promoting family-oriented work.

### Ethics statement

The studies involving human participants were reviewed and approved by the Ethik Kommission der Ärztekammer Hamburg. Participants provided their written informed consent to participate in this study.

### Supplementary Information

**Additional file 1.**

## Data Availability

The datasets generated and/or analysed during the current study are not publicly available due we do not have the consent from the participants and the participating clinical centers, but are available from the corresponding author on reasonable request.

## Abbreviations

ANOVA: Analysis of Variance
CHIMPS: Children of mentally ill parents
CHIMPS-NET: Children of mentally ill parents research network
Ci-chimps: The clinical implementation study of CHIMPS with three implementation interventions
FFMHPQ: Family-Focused Mental Health Practice Questionnaire
FFMHPQ-GV: Family-Focused Mental Health Practice Questionnaire-German Version
ICQ: Implementation Components Questionnaire
ISS: Implementation Satisfaction Scale
M: Mean
MCAR-test: Missing Completely at Random Test
SD: Standard deviation
SPSS: Statistical Package for the Social Sciences
