# Supplementary material for: Different perspectives in psychiatry: how family-oriented are professionals in Germany?
Source: BMC Psychiatry. 2024 Feb 20;24:142. doi: 10.1186/s12888-024-05562-0 (PMC10880271; doi:10.1186/s12888-024-05562-0)
Supplement: Supplementary file 1 — Additional file 1. [file 12888_2024_5562_MOESM1_ESM.pdf]

Manual for the

Family focused mental health practice questionnaire

First Edition

DO NOT COPY, DISTRIBUTE OR CITE WITHOUT PERMISSION FROM THE AUTHORS

Maybery, Goodyear & Reupert, 2012

Appendix One: 53 item version of the Family focused mental health practice questionnaire

Family focused mental health practice questionnaire

(Maybery, Goodyear & Reupert, 2012)

The central aim of this survey is to determine training needs and interests regarding family focused mental health practices. The survey focuses upon mental health worker skill and knowledge, workplace policies and procedures, time, workload and location problems, opportunities for professional development and engagement and confidence issues in relation to working with parents with a mental illness, their families and children. Questions also focus on the capacity and interest that workers have to work with families and children of parent-consumers.

If you decide to participate, the survey will take between 10 and 20 minutes to complete. We are interested in your honest feedback about your activities and your organisation. There is no right or wrong answers.

For each question below please circle the answer (number) that best corresponds with your experience. Please note that the term 'family work' is used generally to describe the process of working with children and families and with mental health consumers about parenting issues. Use the following scale as a guide to answer.

Not applicable	Strongly Disagree	Disagree	Slightly Disagree	Neither agree or disagree	Slightly Agree	Agree	Strongly agree
N/A	1	2	3	4	5	6	7

1	My workplace provides supervision and/or mentoring to support workers undertaking child-related work in regard to their consumer-parents (ws1)	N/A	1	2	3	4	5	6	7
2	In my area we lack services (e.g. other agencies) to refer children to in relation to their parent's mental illness (ie programs for children)(li1)	N/A	1	2	3	4	5	6	7
3	There is no time to work with families or children(tw1)	N/A	1	2	3	4	5	6	7
4	Government policy regarding family focused practice is very clear(pp1)	N/A	1	2	3	4	5	6	7
5	Professional development regarding family focused practice is not encouraged at my work place(pd1)	N/A	1	2	3	4	5	6	7
6	I often receive support from co-workers in regard to family focused practice(cs1)	N/A	1	2	3	4	5	6	7
7	I regularly have family meetings (not therapy) with consumer-parents and their family(fp1)	N/A	1	2	3	4	5	6	7
8	I am not confident working with consumer-parents about their parenting skills(wc1)	N/A	1	2	3	4	5	6	7
9	I don't provide information to the carer and/or family about the consumer-parent's medication and/or treatment(sc1)	N/A	1	2	3	4	5	6	7
10	Many consumer-parents do not consider their illness to be a problem for their children(ei1)	N/A	1	2	3	4	5	6	7

DO NOT COPY, DISTRIBUTE OR CITE WITHOUT PERMISSION FROM THE AUTHORS

11	I am able to determine the developmental progress of the children of my consumer-parents(aic1)	**N/A**	**1**	**2**	**3**	**4**	**5**	**6**	**7**
12	I sometimes wish that I was better able to help consumer-parents, discuss the impact of their mental illness on their children(t1)	**N/A**	**1**	**2**	**3**	**4**	**5**	**6**	**7**
13	I am knowledgeable about how parental mental illness impacts on children and families(sk1)	**N/A**	**1**	**2**	**3**	**4**	**5**	**6**	**7**
14	There are no parent-related programs (e.g. parenting skills) to refer consumer-parents to(sa1)	**N/A**	**1**	**2**	**3**	**4**	**5**	**6**	**7**
15	I am able to determine the level of importance that consumer-parents place on their children maintaining attendance at day to day activities such as school and hobbies (e.g. sport, dance)(c1)	**N/A**	**1**	**2**	**3**	**4**	**5**	**6**	**7**
16	I do not refer children of consumer-parents to child focused (e.g. peer support) programs (other than child and adolescent mental health)(r1)	**N/A**	**1**	**2**	**3**	**4**	**5**	**6**	**7**
17	Working with other health professionals enhances my family-focused practice (ic1)	**N/A**	**1**	**2**	**3**	**4**	**5**	**6**	**7**
18	My workplace does not provide supervision and/or mentoring to support workers undertaking family focused practices (ws2)	**N/A**	**1**	**2**	**3**	**4**	**5**	**6**	**7**
19	Due to location it is difficult to coordinate families and children with the required services(li2)	**N/A**	**1**	**2**	**3**	**4**	**5**	**6**	**7**
20	The workload is too high to do family focused work(tw2)	**N/A**	**1**	**2**	**3**	**4**	**5**	**6**	**7**
21	At my workplace, policies and procedures for working with consumer-parents on family issues are very clear(pp2)	**N/A**	**1**	**2**	**3**	**4**	**5**	**6**	**7**
22	My workplace provides little support for further training in family focused practices(pd2)	**N/A**	**1**	**2**	**3**	**4**	**5**	**6**	**7**
23	In my workplace other workers encourage family focused practice(cs2)	**N/A**	**1**	**2**	**3**	**4**	**5**	**6**	**7**
24	I provide written material (e.g. education, information) about parenting to consumer-parents(fp2)	**N/A**	**1**	**2**	**3**	**4**	**5**	**6**	**7**
25	I am not confident working with families of consumer-parents(wc2)	**N/A**	**1**	**2**	**3**	**4**	**5**	**6**	**7**
26	Rarely do I advocate for the carers and/or family when communicating with other professionals regarding the consumer-parent's mental illnesses (sc2)	**N/A**	**1**	**2**	**3**	**4**	**5**	**6**	**7**
27	Discussing issues for the consumer parent with others (including family) would breach their confidentiality(ei2)	**N/A**	**1**	**2**	**3**	**4**	**5**	**6**	**7**
28	I am able to assess the level of children's involvement in their parent's symptoms or substance abuse(aic2)	**N/A**	**1**	**2**	**3**	**4**	**5**	**6**	**7**
29	I should learn more about how to assist consumer-parents about their parenting and parenting skills(t2)	**N/A**	**1**	**2**	**3**	**4**	**5**	**6**	**7**
30	I do not have the skills to work with consumer-parents about how parental mental illness impacts on children and families(sk2)	**N/A**	**1**	**2**	**3**	**4**	**5**	**6**	**7**
31	There are no family therapy or family counselling services to refer consumer-parents and their families to(sa2)	**N/A**	**1**	**2**	**3**	**4**	**5**	**6**	**7**
32	I am able to determine the level of importance that consumer-parents place on their children maintaining strong relationships with other family members (e.g. other parent, siblings)(c2)	**N/A**	**1**	**2**	**3**	**4**	**5**	**6**	**7**
33	I refer consumer-parents to parent-related programs (e.g. parenting skills)(r2)	**N/A**	**1**	**2**	**3**	**4**	**5**	**6**	**7**

DO NOT COPY, DISTRIBUTE OR CITE WITHOUT PERMISSION FROM THE AUTHORS

34	Children and families ultimately benefit if health professionals work together to solve the family's problems (ic2)	**N/A**	**1**	**2**	**3**	**4**	**5**	**6**	**7**
35	There is time to have regular contact with other agencies regarding families or children or consumer-parents(tw3)	**N/A**	**1**	**2**	**3**	**4**	**5**	**6**	**7**
36	I regularly provide information (including written materials) about mental health issues to the children of consumer-parents(fp3)	**N/A**	**1**	**2**	**3**	**4**	**5**	**6**	**7**
37	Rarely do I consider if referral to peer support program (or similar) is required by my consumer-parent's children(sc3)	**N/A**	**1**	**2**	**3**	**4**	**5**	**6**	**7**
38	The children often do not want to engage with me about consumer parents mental illness(ei3)	**N/A**	**1**	**2**	**3**	**4**	**5**	**6**	**7**
39	I would like to undertake future training to increase my skills and knowledge for working with the children of consumer-parents(t3)	**N/A**	**1**	**2**	**3**	**4**	**5**	**6**	**7**
40	I am not experienced in working with child issues associated with parental mental illness(sk3)	**N/A**	**1**	**2**	**3**	**4**	**5**	**6**	**7**
41	I am not able to determine the level of importance that consumer-parents place on their children maintaining strong relationships with others outside the family (e.g. other children/peers, school)(c3)	**N/A**	**1**	**2**	**3**	**4**	**5**	**6**	**7**
42	Team-working skills are essential for all health professionals providing family-focused care (ic3)	**N/A**	**1**	**2**	**3**	**4**	**5**	**6**	**7**
43	I often consider if referral to parent support program (or similar) is required by consumer-parents(fp4)	**N/A**	**1**	**2**	**3**	**4**	**5**	**6**	**7**
44	I would like to undertake training in future to increase my skills and knowledge about helping consumer-parents with their parenting(t4)	**N/A**	**1**	**2**	**3**	**4**	**5**	**6**	**7**
45	I am skilled in working with consumer-parents in relation to maintaining the wellbeing and resilience of their children(sk4)	**N/A**	**1**	**2**	**3**	**4**	**5**	**6**	**7**
46	I want to have a greater understanding of my profession in a healthcare team approach to working with children and families (ic4)	**N/A**	**1**	**2**	**3**	**4**	**5**	**6**	**7**
47	I provide education sessions for adult family members (e.g. about the illness, treatment)(fp5)	**N/A**	**1**	**2**	**3**	**4**	**5**	**6**	**7**
48	I am not confident working with children of consumer-parents(wc3)	**N/A**	**1**	**2**	**3**	**4**	**5**	**6**	**7**
49	I am knowledgeable about the key things that consumer-parents could do to maintain the wellbeing (and resilience) of their children(sk5)	**N/A**	**1**	**2**	**3**	**4**	**5**	**6**	**7**
50	I am not able to determine the level of attachment/bond that consumer-parents have with their children (pmi1)	**N/A**	**1**	**2**	**3**	**4**	**5**	**6**	**7**
51	I am not knowledgeable about the key parenting issues for consumer-parents (pmi2)	**N/A**	**1**	**2**	**3**	**4**	**5**	**6**	**7**
52	I am skilled in working with consumer-parents regarding their parenting (pmi3)	**N/A**	**1**	**2**	**3**	**4**	**5**	**6**	**7**
53	I don't feel confident to counsel consumer-parents about parenting and their mental health problem (pmi4)	**N/A**	**1**	**2**	**3**	**4**	**5**	**6**	**7**

Thank you for completing the questionnaire

Acknowledgements: the development of this survey relied upon the considerable wisdom and input of the Victorian (Australia) FaPMI coordinators. They provided their wisdom regarding the wording and face validity of items and made data collection happen. We also thank Associate Professor Kym Foster, Kylie Eddy and Elizabeth Fudge for their collaboration in developing the inter professional items.

DO NOT COPY, DISTRIBUTE OR CITE WITHOUT PERMISSION FROM THE AUTHORS
